# Editorial: Nucleic Acid-Associated Inflammation

**DOI:** 10.3389/fimmu.2021.791580

**Published:** 2021-10-27

**Authors:** Nadine Laguette, Christelle Langevin, David Olagnier, Vincenzo Torraca, Claire Vanpouille-Box, Eloi R. Verrier

**Affiliations:** ^1^Institut de Génétique Humaine, CNRS, Université de Montpellier, Molecular Basis of Inflammation Laboratory, Montpellier, France; ^2^Université Paris-Saclay, INRAE, IERP UE0907, Jouy-en-Josas, France; ^3^Department of Biomedicine, Aarhus University, Aarhus, Denmark; ^4^Department of Infection Biology, London School of Hygiene and Tropical Medicine, London, United Kingdom; ^5^Department of Radiation Oncology, Weill Cornell Medical College, New York, NY, United States; ^6^Sandra and Edward Meyer Cancer Center, New York, NY, United States; ^7^Université de Strasbourg, Inserm, Institut de Recherche sur les Maladies Virales et Hepatiques UMR_S1110, Strasbourg, France

**Keywords:** inflammation, nucleic acid sensing, DNA, RNA, type I interferon

Inflammatory responses are essential to maintain organismal homeostasis and are typically induced when autonomous defenses are impaired. For instance, inflammation can be engaged in response to tissue damage or exposure to a variety of agents, including pathogenic microorganisms such as viruses and bacteria as well as toxins and xenobiotics. Sensing of inflammatory agents largely relies on the recognition of pathogen-associated molecular patterns (PAMPs), including microbe-associated molecular patterns (MAMPs), through pattern recognition receptors (PRRs) (1). Endogenous factors that are present in an aberrant location (*e.g.* cytoplasmic nucleic acid or extracellular ATP), and/or in a disproportionate abundance, can also activate PRRs and are commonly known as damage-associated molecular patterns (DAMPs) (2). Upon PAMPs or DAMPs recognition, PRRs coordinate an early response of the host to endogenous or exogenous threats that prime the host for the activation of adaptive immunity (Okude et al.) (3).

Given the central role of PRRs in the control of invading pathogens and endogenous threats, it is not surprising that genetic or etiological alterations of inflammation foster a wide range of human pathologies. Underscoring this concept, the persistent dysregulation of nucleic acid-associated inflammatory pathways has been associated with the development of chronic liver diseases. The two main etiology agents that are linked with these liver pathologies are hepatitis B virus (HBV) and hepatitis virus C (HPC). These viruses have distinct genomes and viral life cycles but can both repress innate anti-viral defenses through common mechanisms. These strategies are being discussed in our Research Topic “*Nucleic Acid-Associated Inflammation*” by Roca Suarez et al., as well as Xu et al.

Innate immunity largely relies on the recognition of evolutionarily conserved structures that can be identified *via* comparative analyses of innate immune responses, from biological models (zebrafish and mouse) to human. This underscores the existence of tissue- and species- specificities, and is discussed in six reviews of the Research Topic which cover various aspects, ranging from the role of transposable elements to the limitations of *in vivo* models and provides cues towards the development of high content therapeutic strategies in relevant physiological models (Jacquet et al., Jami et al., Magnani et al., Rutherford et al., Sullivan et al., Verrier et al.).

However, despite major advances in the field of innate immunity to identify the pathways involved in the onset of cytokine production in response to immune-stimulatory nucleic acids, there are still many open questions. Specifically, how these signalling pathways are regulated in respect to various nucleic acid substrates and tissue insults. In this special Research Topic, key opinion leaders in the field offer an overview of (1) the major molecular and cellular aspects of nucleic acid sensing across species; (2) the complexity of innate and adaptive immune responses and their key role in the maintenance of tissue homeostasis; and (3) the intricate connections between deregulated nucleic acid sensing machinery and human disease. For instance, reviews by Santa et al. and by Kumar give a comprehensive overview of the regulatory circuitries of nucleic acid-sensing pathways.

Even though nucleic acid-associated inflammation is the first line of defense of the host, activation of innate immunity is not always guaranteed. Indeed, microbes and malignant cells have developed a variety of strategies to prevent inflammation, in order to counteract the host response or escape the induction of anti-tumor immunity (4). Supporting this concept, evidence demonstrates that nucleic acid sensing is critical to generate radiation-induced anti-tumor immunity (5). Such responses result from the recognition of mitochondrial DNA in irradiated tumor cells by the cyclic GMP-AMP synthase (cGAS) sensor of cytosolic DNA (6). Since then, efforts in uncovering the adjuvanticity of radiation therapy from multiple types of ionizing radiations have pointed towards the role of DNA damage response and cytosolic DNA detection in priming anti-tumor responses. Some of the current views of how genotoxic stress crosstalk with innate immune responses and anti-tumoral immunity are summarized in reviews by Constanzo et al. and by Taffoni et al.

Regulating abnormal nucleic acid sensing is emerging as a potent strategy against inflammatory diseases. Thus, PRRs and their downstream effectors have become attractive targets for the identification of biomarkers and the development of therapeutic agents with broad-range efficacy against inflammatory disorders. Review by Hemphill et al. from this Research Topic is discussing the therapeutic potential of the three-prime repair exonuclease 1 (TREX1) targeting as a novel immunotherapy strategy against cancer.

In conclusion, each one of the reviews and articles presented in *Nucleic Acid-Associated Inflammation* focuses on one specific aspect of nucleic acid sensing, encompassing signaling, regulation, interspecies specificities, and pathology relevance. The Research Topic is equally addressed to expert investigators who may wish to extend their knowledge on inflammation, innate and nucleic acid immunity, and to newcomers to this exciting and quickly progressing field of investigation.

## Author Contributions

All authors wrote the manuscript. All authors contributed to manuscript revision, read, and approved the submitted version.

## Funding

NL is supported by the European Research Council (ERC-Stg CrIC: 637763, ERC-PoC DIM-CrIC: 893772), “LA LIGUE pour la recherche contre le cancer” and the “Agence Nationale de Recherche sur le SIDA et les Hépatites virales” (ANRS: ECTZ117448). CL is supported by the EU INFRAIA project VetBioNet (EU H2020 project 731014) and received institutional support from INRAE. The INRAE Infectiology of Fishes and Rodents Facility (IERP-UE907, Jouy-en-Josas Research Center, France) belongs to the National Distributed Research Infrastructure for the Control of Animal and Zoonotic Emerging Infectious Diseases through *In Vivo* Investigation (EMERG’IN DOI: 10.15454/90CK-Y371). DO is supported by the Lundbeck Foundation (R335-2019-2138), a young talented cancer researcher grant from Kræftens Bekæmpelse (R279-A16218), the Brødrene Hartman Fond, the Hørslev Fond, the fabrikant Einer Willumsens mindelegat, and the Eva og Henry Fraenkels Mindefond. VT is supported by an LSHTM/Wellcome Institutional Strategic Support Fund (ISSF) Fellowship (204928/Z/16/Z). CV-B is supported by a startup grant from the Dept. of Radiation Oncology at Weill Cornell Medicine (New York, US) and by WCM Prostate Cancer SPORE Development Research Program Award. EV is supported by ANRS (ECTZ104527), LabEx HepSYS (ANR-10-LAB-28), and the French National Research Agency (ANR-21-CE15-0035-01).

## Conflict of Interest

The authors declare that the research was conducted in the absence of any commercial or financial relationships that could be construed as a potential conflict of interest.

## Publisher’s Note

All claims expressed in this article are solely those of the authors and do not necessarily represent those of their affiliated organizations, or those of the publisher, the editors and the reviewers. Any product that may be evaluated in this article, or claim that may be made by its manufacturer, is not guaranteed or endorsed by the publisher.
